# The Potentials of Egyptian and Indian Granites for Protection of Ionizing Radiation

**DOI:** 10.3390/ma14143928

**Published:** 2021-07-14

**Authors:** Mohamed Elsafi, M. F. Alrashedi, M. I. Sayyed, Ibrahim F. Al-Hamarneh, M. A. El-Nahal, Mostafa El-Khatib, Mayeen Uddin Khandaker, Hamid Osman, Ahmad El Askary

**Affiliations:** 1 Physics Department, Faculty of Science, Alexandria University, Alexandria 21511, Egypt; matab.1413@gmail.com; 2 Physics Department, College of Science and Arts, Al-Qassim University, Buraydah 52571, Saudi Arabia; 3 Department of Physics, Faculty of Science, Isra University, Amman 11622, Jordan; dr.mabualssayed@gmail.com; 4 Department of Nuclear Medicine Research, Institute for Research and Medical Consultations, Imam Abdulrahman bin Faisal University, Dammam 31441, Saudi Arabia; 5 Department of Physics, Faculty of Science, Al-Balqa Applied University, Al-Salt 19117, Jordan; hamarnehibrahim@gmail.com; 6 Department of Environmental Studies, Institute of Graduate Studies and Research, Alexandria University, Alexandria 21526, Egypt; Igsr.nahalmoh@alexu.edu.eg; 7 Basic Sciences Department, Faculty of Engineering, Pharos University in Alexandria, Alexandria 21526, Egypt; mostafa.elkhatib@pua.edu.eg; 8 Centre for Applied Physics and Radiation Technologies, School of Engineering and Technology, Sunway University, Bandar Sunway 47500, Selangor, Malaysia; 9 Department of Radiology, College of Applied Medical Sciences, Taif University, Taif 21944, Saudi Arabia; ha.osman@tu.edu.sa; 10 Department of Clinical Laboratory Sciences, College of Applied Medical Sciences, Taif University, Taif 21944, Saudi Arabia; a.elaskary@tu.edu.sa

**Keywords:** granite, gamma radiation, shielding properties, experimental data, red aswani granite, effective shielding

## Abstract

This paper aims to study the radiation shielding characteristics and buildup factor of some types of granite in Egypt. The mass attenuation coefficient (MAC) for three types of granite (gandola, white halayeb, and red aswani) was experimentally determined, and the experimental results were validated by XCOM software. The relative deviation between the two methods does not exceed 3% in all discussed granite samples, which means that MAC calculated through the experimental and XCOM are in suitable agreement. The effective atomic number (Z_eff_) varies from 13.64 to 10.69, 13.68 to 10.59, and 13.45 and 10.66 for gandola, white halayeb, and red aswani, respectively. As well as the equivalent atomic number (Z_eq_) was calculated in a wide range of energy to deduce the exposure (EBF) and energy absorption (EABF) buildup factors for the studied granite materials. The linear attenuation coefficient (LAC), half-value layer (HVL), mean free path (MFP) were calculated at each investigated energy and showed that the most effective shielding ability at high energy was red aswani, while at low energy, the shielding ability was nearly constant for studied granites. The present study forms the first endeavor to obtain the radiation shielding properties of the studied materials to be used in practical applications.

## 1. Introduction

Radiation is currently used in the medical field, for agricultural purposes, in energy generation, in food processing, and many more. As the years continue to pass, the number of applications that use radiation to function increases, as well as its potential benefits [1,2,3]. Over the past several decades, immense progress has been conducted in using radiation as a treatment to fight cancerous cells and in radiology. These numerous applications demonstrate the necessity of radiation in our daily lives. Despite these benefits, it is important to keep in mind the potentially dangerous nature of radiation [4,5,6].

Ionizing radiation, specifically, is radiation that has sufficient energy to detach electrons from atoms, which can cause great damage if the human body is exposed to these photons for a long period of time. For example, long-term exposure to gamma radiation can lead to permanent tissue damage, acute radiation poisoning, cancer, and death in extreme cases. Thus, to prevent workers and patients who are exposed to radiation from these potential side effects, radiation shields are typically used as a protective measure [7,8,9,10].

Radiation shields are defined as any material placed between the radiation source and the human body that is used to absorb incoming photons are reducing the level of radiation to safe enough levels. Depending on the specific application and conditions that the radiation is being used in, different types of radiation shielding materials are used [11,12]. For example, in the examples where transparency is an essential component needed, glasses doped with heavy metal oxides are typically used. In other cases, however, glasses may not be the most effective material, which is why specific details of the environment of the radiation source must be known [13,14,15]. Other commonly used radiation shielding materials that are currently being researched include alloys, composites, and concrete [16,17,18].

Construction materials such as granite are also undergoing investigation to determine their radiation shielding capability [19]. Granite is an igneous rock that is formed from magma. Granite is mostly found at a depth of greater than 1.5 km. It is commonly used for temperature isolation, for its high durability, and for its aesthetic purposes as a decorative material. The term ‘granite’ is used to describe all igneous rock types used as building materials, so other igneous rocks will be considered as ‘granite’ in this study. Previous studies have mainly analyzed the structural properties of granite samples, but more research is needed to evaluate their radiation shielding capabilities [20,21,22]. Since the granites already exist as common building material or decorative tiles in various dwellings, thus this study investigates the capability of the common existence of granites in the purpose of gamma radiation shielding.

This study aims to determine the radiation shielding properties for different kinds of granite samples used in construction and as building materials to obtain a comprehensive understanding of the characteristics. To evaluate the attenuation capability of the samples, their mass attenuation coefficient (MAC) was experimentally determined, alongside other vital parameters. By analyzing these parameters, the most and least effective samples can be determined across a wide range of energies.

## 2. Materials and Methods

### 2.1. Samples Preparation

Three types of Egyptian granites were selected among the available and low-cost granite tiles in the local Egyptian market. The samples were shaped into uniform slabs of dimensions of 5 cm length, 5 cm width, and 1 cm thickness to facilitate the gamma radiation attenuation measurements. Ten samples are produced of each granite kind. Another ten samples in the form of homogeneous powder were produced and ground, and the final powder samples weight was 100 g with an accuracy of 0.001 g to be analyzed by the energy-dispersive spectrometer (EDX) technique (JEOL Ltd, Tokyo, Japan).

### 2.2. Sample Characterization

Measuring the average density and identifying the chemical structure of each granite type is essential in the present study in order to determine the mass attenuation coefficients of examined samples; the density can be measured directly by determining the sample volume and mass accurately due to sampling solidity and shape uniformity. The chemical compositions were determined by using energy-dispersive X-ray analysis by using the energy-dispersive spectrometer of the scanning electron microscope unit at Alexandria University in Egypt, Model JEOL-JSM-6360LA. Three regions of the sample were scanned, and the average composition was calculated so that the chemical composition of each granite sort can be determined accurately. The trace elements were neglected, and the estimated averages of the chemical compositions of different types of granites are given in Table 1.

### 2.3. Gamma Rays Attenuation Measurements

**Figure 1:**
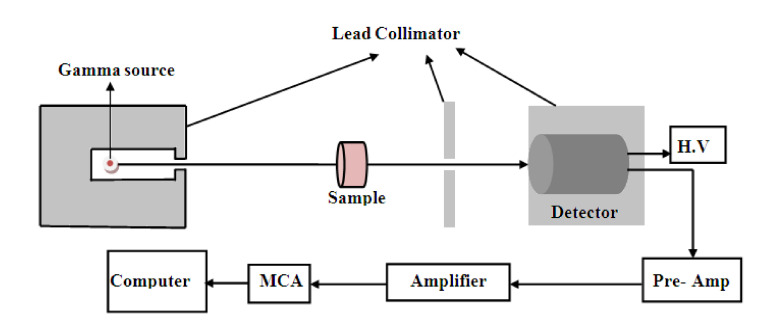
The experimental setup for the determination of the mass attenuation coefficients.

The narrow beam arrangement, as illustrated in Figure 1, was used to measure the attenuation coefficients of the investigated samples. The effectiveness of the narrow beam method in determining the attenuation parameters has been proven in many previous works of literature [23,24]. The initial intensities (without introducing the attenuator sample) of gamma rays emitted from different radioactive sources with various energy were detected by a hyper pure germanium detector, and then the sample was introduced into the path of gamma radiation as an attenuator. Finally, the attenuated intensities were recorded. The accurate and precise radiation measurement requires well energy and efficiency calibration performed before the measuring process. The radioactive point sources used in the detector calibration were Am-241 (59.5 keV), Cs-137 (661.7 keV), and Co-60 (1173.2 keV and 1332.5 keV). The counting time was constant and long enough to reduce the uncertainty to be 1% or less [25,26,27,28]. The used radioisotopes were purchased from Physikalisch-Technische Bundesanstalt PTB in Braunschweig and Berlin, and the radiation parameters of radiation sources used in the present study are tabulated in Table 2. The recorded spectra were analyzed by the Genie 2000 data acquisition and analysis software made by Canberra.

### 2.4. Shielding Parameter Calculations

The MAC can be determined from the well-known Beer–Lambert’s law [29] as follows:

(1) $$\mathrm{MAC}=\frac{1}{x\;\rho }\ln\left(\frac{I_{0}}{I}\right)$$

where *I*_0_ and *I* are the incident and transmitted intensities, respectively, passing through a target material of thickness *x*, and ρ is the density of the granite sample. The intensity of the gamma-ray line represents the count rate or the peak area per unit time, which were analyzed using the Genie 2000 software. In addition, by knowing the initial and transmitted intensities, the transmission factor TF can experimentally be calculated at varying sample thicknesses.

The LAC is affected by the density of the absorber, so to calculate the LAC, the MAC must be multiplied by the density of the measured sample. The HVL is the thickness needed to reduce the intensity of the incoming photons by 50%, and its equation is the following [30]:

(2) $$\mathrm{HVL}=\frac{\ln2}{LAC}$$

The mean free path MFP is the average distance that a photon travels between two successive interactions and is described by the following equation [30]:

(3) $$\mathrm{MFP}=\frac{1}{LAC}$$

## 3. Results and Discussion

The chemical analysis for the three granite samples was performed, and their average chemical composition is presented in Table 1. The data presented in Table 1 reveal the similarity in the chemical composition of the three granites with high contents of silica, SiO_2_ (ranging from ~74% to ~78%), and alumina Al_2_O_3_ (ranging from ~13% to ~15%). The density of gandola (G.G) and red aswani (G.RA) samples are almost equal and higher than that of white halayeb (G.WH) sample. The chemical analysis of the samples also showed that G.G and G.WH samples have small amounts of transition metals (Ti, Cr, Mn, Fe, and Cu), whereas the G.RA sample has none of these elements.

**Figure 2:**
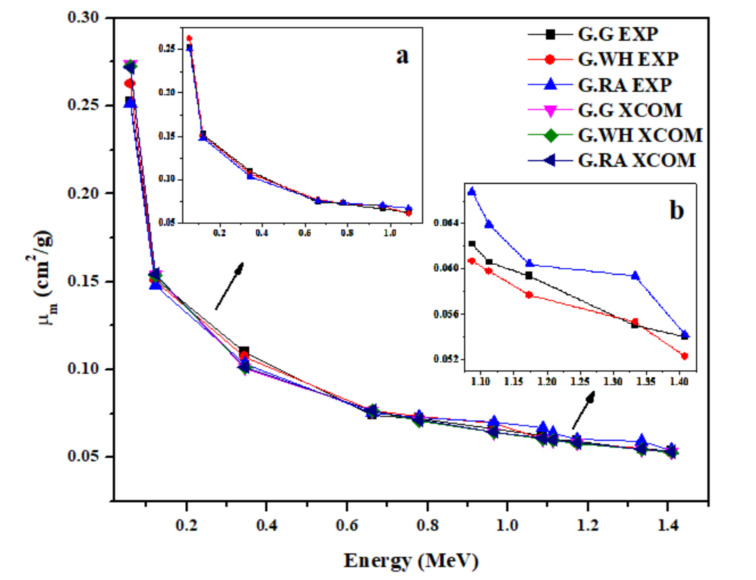
Variation of the experimental and theoretical values of the mass attenuation coefficient (μ_m_) with photon energy for the granite samples in the energy range 0.015 to 15 MeV. Insets (**a**,**b**) to the figure show the variation of the experimentally obtained μ_m_ in the low and high energy ranges, respectively.

The experimental values of mass attenuation coefficients (μ_m_) for the investigated granites at the designated photon energies are given in Table 3**.** The validity of the transmission geometry used for determining μ_m_ of the present granite samples was investigated by comparing the experimental μ_m_ values with the theoretical ones that were calculated by WinXCOM [31]. From Table 3, it could be concluded that the agreement is quite satisfactory between the experimental and theoretical μ_m_ values as the deviation percents given in the table are less than 5% in most cases. However, larger deviations up to about 10% were observed in a few cases. In addition, the experimental and theoretical values of μ_m_ for granite samples at selected energies between 0.0595 and 1.4080 MeV are plotted in Figure 2.

It can be seen from Figure 2 and Table 3 that μ_m_ values tend to be maximized (in the range of 0.2512 to 0.2627 cm^2^/g) at low gamma-ray energies (0.0595 MeV) and decrease as energy increases for all granite samples. The decrease in μ_m_ is considerable in lower photon energies (between 0.0595 and 0.3443 MeV), whereas the change in μ_m_ is moderate at higher energies (between 0.6617 and 1.4080 MeV). The variation in μ_m_ coefficients with energy can be explained based on the cross-section for the different photon interaction mechanisms. At low energies (between 0.0595 and 0.3443 MeV), the cross-section of the photoelectric absorption depends on the atomic number as Z^4−5^, which results in higher values of μ_m_, whereas the cross-section of this process depends on the photon energy as 1/E^3.5^, which causes a higher decreasing rate in μ_m_. At intermediate energies (between 0.6617 and 0.9641 MeV) at which the Compton scattering is dominant, the cross-section of the interaction process depends linearly on the atomic number and inversely on the photon energy; therefore, a slight decrease in μ_m_ values was observed. In the high energy range (between 1.0860 and 1.4080 MeV), μ_m_ values are almost constant for the three granite samples, which indicates that the present granites have no significant effect on the attenuation of γ-ray photons at this range of energy. This energy dependence overlaps with other findings reported for natural marbles [32], for ceramics [33], and for rocks including granite [34] in the same energy range.

Seen also from Figure 2 the dependence of μ_m_ on the chemical composition and, accordingly, on the density of granite. Inset a to Figure 2 shows that μ_m_ coefficients at low energies (0.0595–0.9641 MeV) are almost the same for the three samples due to the similarity in their chemical composition. On the other hand, inset b to Figure 2 shows shows that μ_m_ coefficients are influenced by density at high energies (1.0860–1.4080 MeV). In this energy range, μ_m_ values of the highest density sample (G.RA) is maximum while those of the lowest density sample (G. WH) are minimized. This indicates that the shielding performance of granite samples improves with increasing density, especially at high energies.

**Figure 3:**
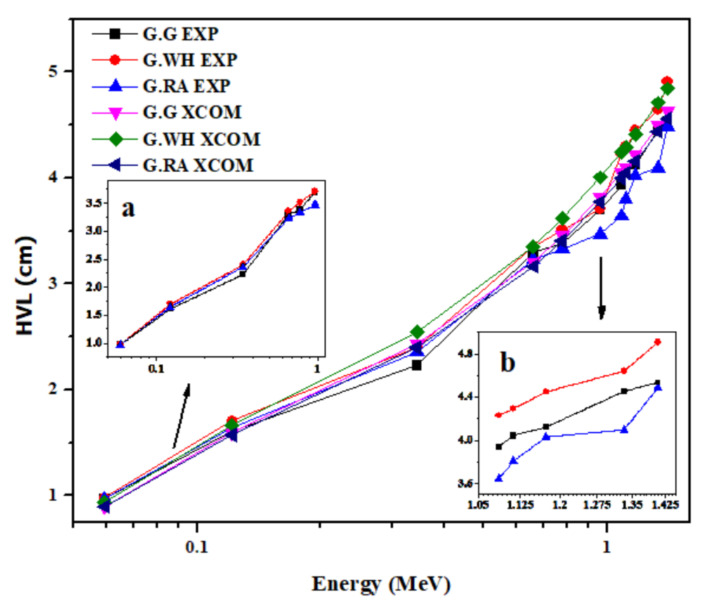
Variation of the experimental and theoretical values of half-value layer (HVL) with photon energy for the granite samples in the energy range 0.015 to 15 MeV. Insets (**a**,**b**) to the figure show the variation of the experimentally obtained HVL in the low and high energy ranges, respectively.

**Figure 4:**
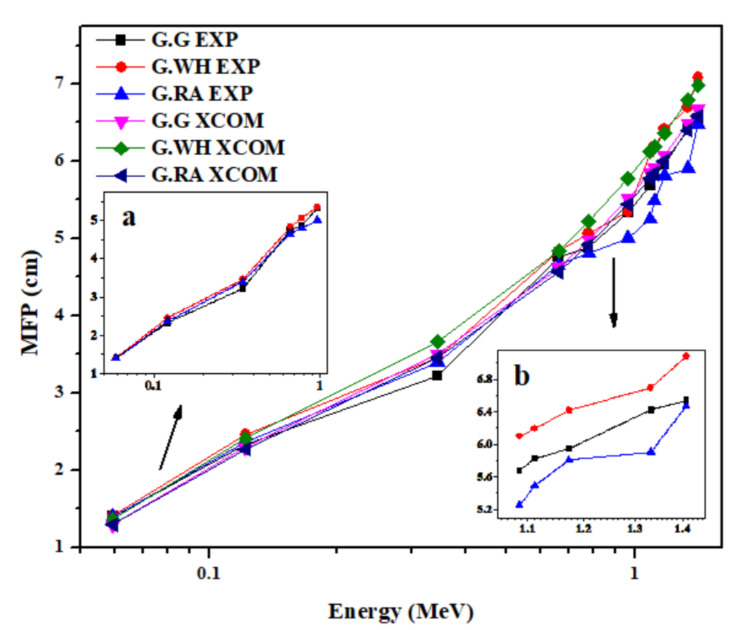
Variation of the experimental and theoretical values of the mean free path (MFP) with photon energy for the granite samples in the energy range 0.015 to 15 MeV. Insets (**a**,**b**) to the figure show the variation of the experimentally obtained MFP in the low and high energy ranges, respectively.

The important parameters that can be used as a measure of the shielding effectiveness of a material are the half-value layer (HVL) and mean free path (MFP). The lower the values of HVL and MFP of a material, the better its capability in lessening gamma radiation. Figure 3 and Figure 4 exhibit the variation of the experimental and theoretical values of HVL and MFP, respectively, on the photon energy. From these two figures, the agreement is evident between the experimental and theoretical values of HVL and MFP. Shown also in Figure 3 and Figure 4 that both HVL and MFP increase as energy increases, which indicates that the shielding performance of the granites reduces down to about 80% as the gamma photon energy lowers from 0.0595 to 1.4080 MeV. Moreover, the insets to Figure 3 and Figure 4 elucidate that the attenuation of γ-ray photons is highly dependent on the density of granite, especially at high energies, at which the highest density sample (G.RA) has relatively lower values of HVL and MFP than the other two less-dense samples. However, the apparent disparity in reducing the intensity of the high-energy γ-ray photons can be compensated for the low-density granites by increasing their thickness, which is crucial in radiation shielding applications. The effect of density on the granite radiation shielding competence obtained in this study fairly matches with the findings of Sayyed et al., 2018 [33] for ceramics and with the findings of Obaid et al., 2018 [34] for marble.

**Figure 5:**
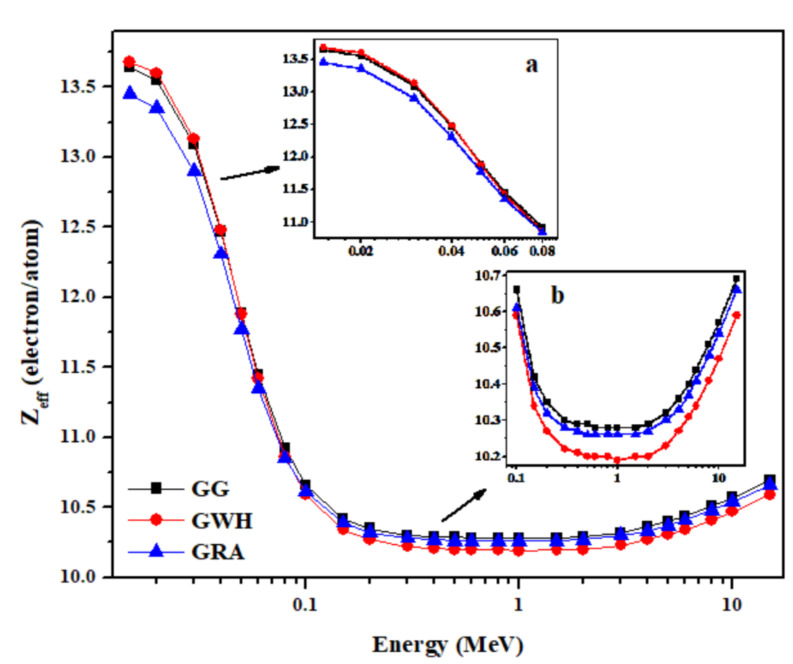
Variation of the calculated values of effective atomic number (Z_eff_) with photon energy for the granite samples in the energy range 0.015 to 15 MeV. Insets (**a**,**b**) to the figure show the variation of the experimentally obtained Z_eff_ in the low and high energy ranges, respectively.

Table 4 presents the calculated effective atomic number (Z_eff_) of granite samples within an energy range of 0.015 to 15 MeV, whereas Figure 5 illustrates the variation by photon energy of Z_eff_ for all interactive processes in the granites. Initially, Z_eff_ is observed to gain maximum values (13.45–13.68) and remains almost constant in the lower energy range (0.015–0.02 MeV) due to the dominance of the photoelectric interaction. As energy increases up to 1 MeV, Z_eff_ decreases sharply to minimum values (10.19–10.28), which indicates that the Compton scattering process takes over. In the intermediate energy range (0.2–2 MeV), almost constant values of Z_eff_ have been observed due to the dominance of the Compton scattering process, whose cross-section is purely energy-dependent and almost independent of the chemical composition of the granite. In the high energy range (3–15 MeV), the values of Z_eff_ increased slowly with energy due to the dominance of the pair production process whose cross-section is proportional to Z^2^. This trend of the energy dependence of Z_eff_ was observed for several materials such as ceramics [33], rocks including granite and marble [19], and soils [34].

Inset a to Figure 5 shows that Z_eff_ values for G.G and G.WH are almost equal and slightly higher than that for G.RA in the low energy range at which the dominant interaction process is the photoelectric absorption that is known to severely dependent on the atomic number of the absorber medium. Therefore, this minor disparity in Z_eff_ between granite samples is mainly due to the presence of higher Ca content in G.G and G.WH samples in comparison with the G.RA sample. In support of that, the chemical analysis of the granite samples given in Table 1 showed the presence of small amounts of relatively high atomic number transition metals (Ti, Cr, Mn, Fe, and Cu) in G.G and G.WH samples, whereas the G.RA sample has none of these elements. As shown in inset b to Figure 5, the granite samples for intermediate and high energies have nearly equal values of Z_eff_, where Compton scattering and pair production processes are dominant. This is because the probability of interaction for these two mechanisms is less dependent on the chemical composition of materials. These findings show the importance of Z_eff_ in revealing the competence of shielding properties of granite as a function of energy.

**Figure 6:**
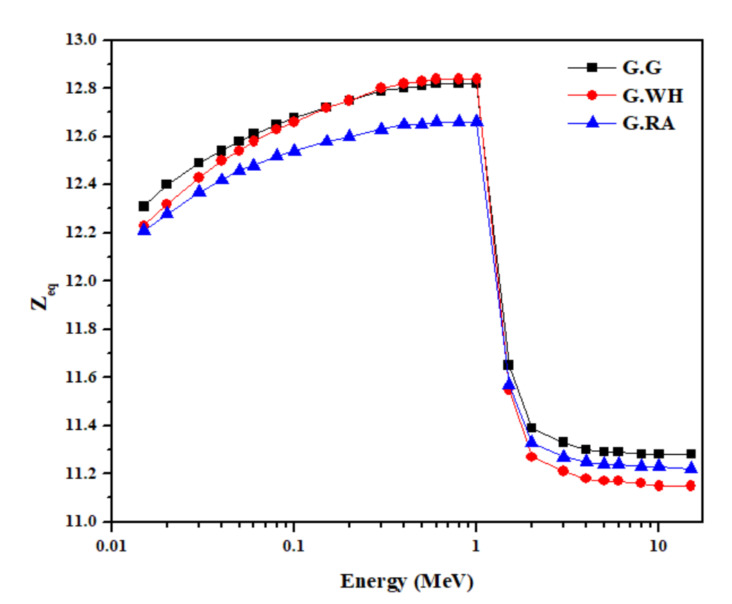
Variation of the calculated values of equivalent atomic number (Z_eq_) with photon energy for the granite samples in the energy range 0.015 to 15 MeV.

Table 4 also presents the calculated equivalent atomic number (Z_eq_) for the granite samples in the energy range 0.015–15 MeV, whereas the variation of Z_eq_ with photon energy is given in Figure 6. For all granites, Z_eq_ tends to be maximum with values between 12.66 and 12.84 at intermediate energies (0.6–1 MeV) and then decreases rapidly to lower values as energy increases due to the dominance of the pair production process. In addition, shown in Figure 6 that Z_eq_ has demonstrated the same energy behavior of Z_eff_ that was presented in Figure 5. Moreover, values of Z_eq_ for G.G and G.WH samples are almost equal and slightly higher than those of the G.RA sample in the low energy range, where the photoelectric absorption is dominant. Since the best shielding material is the one that has high Z_eq_, therefore, G.G and G.WH samples can be considered better in shielding of low gamma-ray energies than the G.RA sample. However, as the energy increases to 1 MeV and above, the G.G sample has better-shielded capabilities than the other two samples, as can be observed from Figure 6.

**Figure 7:**
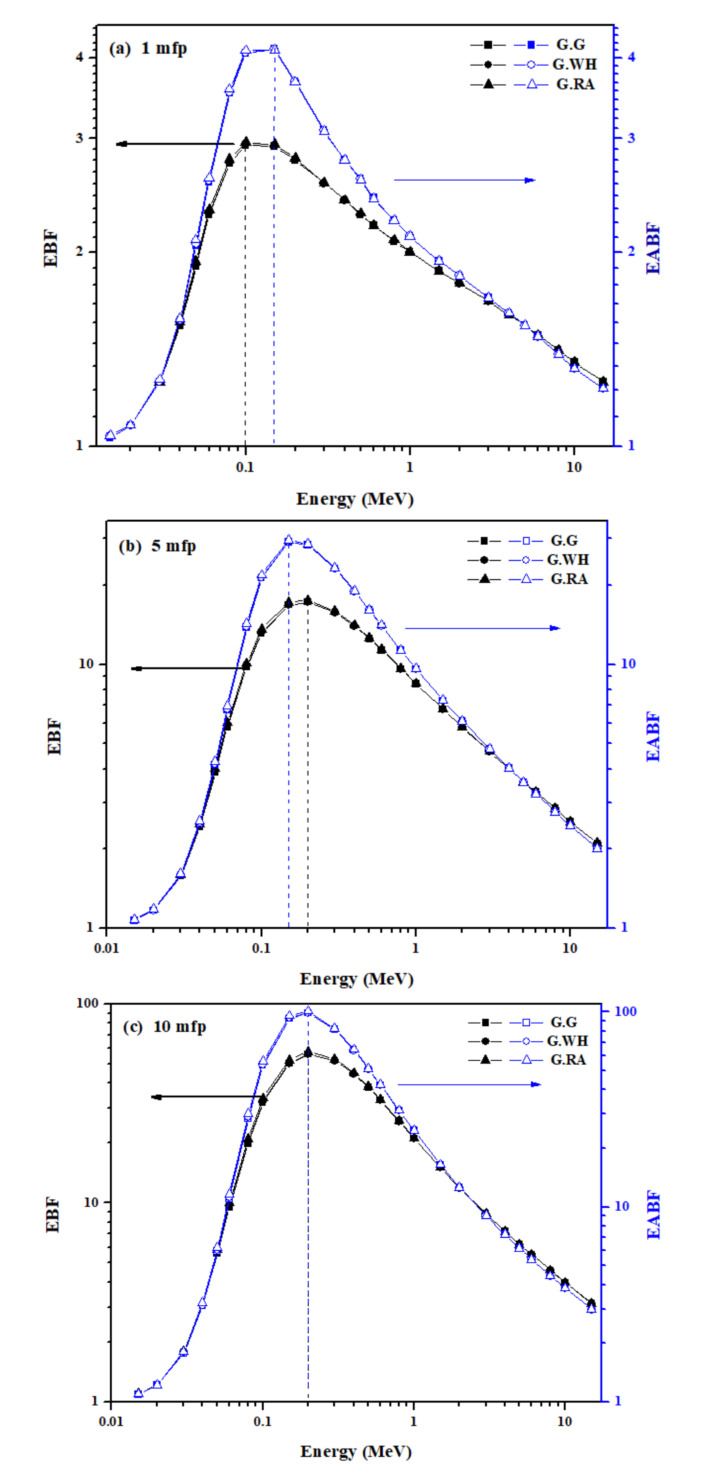

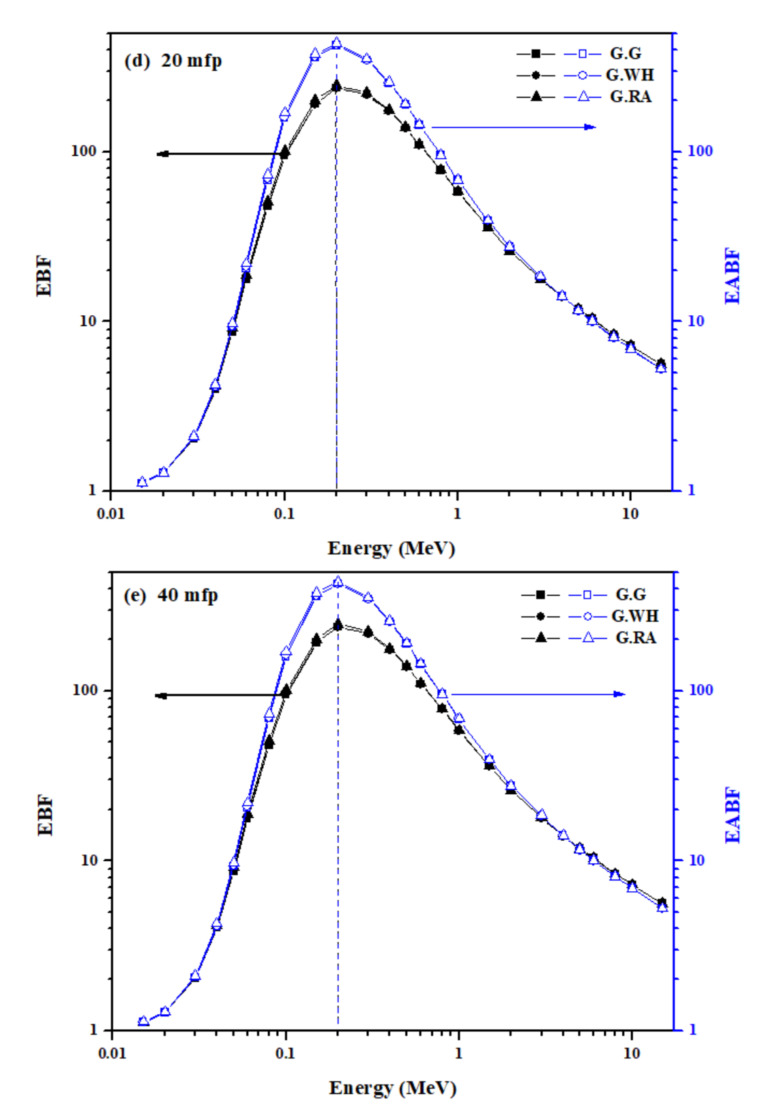
Variation of the calculated values of exposure buildup factor (EBF) and energy absorption buildup factor (EABF) with photon energy for the granite samples in the energy range 0.015 to 15 MeV at the depth penetration (**a**): 1 mfp; (**b**): 5 mfp; (**c**): 10 mfp; (**d**): 20 mfp, and (**e**): 40 mfp.

Figure 7a–e shows the variation with a photon energy of the exposure buildup factor (EBF) and the energy absorption buildup factor (EABF) for the granite samples at penetration depths of 1, 5, 10, 20, and 40 mfp, respectively. These figures illustrate that EBF and EABF increase rapidly with increasing energy and gain their maximum values in the medium energy range, then decrease rapidly with further increasing the energy up to 15 MeV. The energy dependence of EBF and EABF can also be explained based on the dominance of different partial photon interaction mechanisms in the studied energy regions. In the low energy range where the photoelectric effect is dominant, the number of completely absorbed or removed photons is maximized; thus, EBF and EABF show minimum values close to one (i.e., no scattering buildup) for all granite samples. As the photon energy increases, the Compton scattering process starts to dominate, causing more multiple Compton scattering events and, thus, the values of EBF and EABF increase. In the high energy range, the pair production process starts to take over and, hence the values of EBF and EABF reduce again.

As the penetration depth of the granites increases, the thickness of the interacting material increases, which in turn causes the scattering events within the granites to increase and, hence values of EBF and EABF become higher as shown in Figure 7a–e. For the penetration depth 1 mfp, EBF and EABF values for the investigated granites vary from 1.03 to 2.96 and from 1.03 to 4.13, respectively. For the penetration depth 5 mfp, EBF and EABF values for the same granites lies between 1.07–17.5 and 1.07–29.6, respectively, whereas for the penetration depth 10 mfp, EBF and EABF range from 1.09 to 57.6 and 1.09 to 101.0, respectively. For the highest penetration depths of 20 and 40 mfp, both EBF and EABF show their maximum values of 238 and 438, respectively. In addition, it is observed that the granite sample with the lowest Z_eq_ value (i.e., G. RA) dominates the maximum EBF and EABF values, whereas the sample of the highest Z_eq_ value (i.e., G. G) dominates the minimum EBF and EABF values. From these findings, it can be concluded that the G.G granite sample has slightly more shielding effectiveness for gamma-ray energies, whereas the G.RA granite sample is weak in gamma-ray shielding. Finally, Figure 7a–e shows that the maximum EBF and EABF values exhibited a slight shift in energy from 0.1 to 0.2 MeV as the penetration depth increased. Overall, this study reveals that the granites can be considered as a candidate for the purpose of gamma radiation shielding in various dwelling structures but yet to be deployed in real applications.

## 4. Conclusions

This study evaluated the radiation shielding properties and buildup factor of some types of granite in Egypt. The mass attenuation (MAC) for three types of granite was experimentally determined, and the experimental results were validated by XCOM software. The MAC calculated through the experimental and XCOM are in suitable agreement. The linear attenuation coefficient, half-value layer, and mean free path were calculated at each investigated energy and showed that the most effective shielding ability at high energy was red aswani, while at low energy, the shielding ability nearly constant for studied granites. The effective atomic number varies from 13.64 to 10.69, 13.68 to 10.59, and 13.45 to 10.66 for gandola, white halayeb, and red aswani, respectively. As well as the equivalent atomic number was calculated in a wide range of energy, and the EBF and EABF were calculated for the studied granite materials at different mean free paths. The results of EBF and EABF showed the same values for studying granite samples with different energies.

## Figures and Tables

**Table 1:** The average chemical composition of the investigated granites.

Molecular Composition	Average Mass %		
	Granite Gandola G.G ρ = 2.83 g/cm^3^	White Halayeb G.WH ρ = 2.70 g/cm^3^	Red Aswani G.RA ρ = 2.85 g/cm^3^
Al_2_O_3_	12.86	14.99	14.61
SiO_2_	77.92	74.19	77.54
K_2_O	4.57	-	4.70
CaO	3.26	4.22	3.03
TiO_2_	0.36	0.24	-
Cr_2_O_3_	0.07	-	-
MnO	0.06	-	-
CuO	0.10	-	-
Na_2_O	-	4.21	-
FeO	-	1.55	-

**Table 2:** The radioisotopes point source parameters.

PTB Nuclide	Energy keV	Emission Probability	Activity kBq	Reference Date	Uncertainty
Am-241	59.52	35.90	259.0	1.June 2009	±2.6
Cs-137	661.66	34.10	385.0		±4.0
Eu-152	121.78	28.40	290.0		±4.0
	244.69	26.60			
	344.28	14.00			
	964.13	20.87			
	1408.10	85.21			
Co-60	1173.23	99.90	212.1		±1.5
	1332.50	99.98			

**Table 3:** Mass attenuation coefficient (μ_m_) for the granite sample.

E (MeV)	G.G			G.WH			G.RA		
	EXP	XCOM	% Dev.	EXP	XCOM	% Dev.	EXP	XCOM	% Dev.
0.0595	0.2625	0.2735	−4.02	0.2627	0.2725	−3.60	0.2612	0.2718	−3.90
0.1218	0.1519	0.1539	−1.30	0.1507	0.1539	−2.08	0.1481	0.1545	−4.14
0.3443	0.10229	0.1008	1.48	0.102	0.1009	1.09	0.1032	0.1015	1.67
0.6617	0.0742	0.0764	−2.88	0.0766	0.0765	0.13	0.0753	0.0769	−2.08
0.7789	0.0724	0.0709	2.12	0.0731	0.071	2.96	0.073	0.0714	2.24
0.9641	0.0662	0.0641	3.28	0.0632	0.0641	−1.40	0.0621	0.0645	−3.72
1.086	0.0622	0.0604	2.98	0.0607	0.0605	0.33	0.0618	0.0608	1.64
1.112	0.0606	0.0597	1.51	0.0598	0.0598	0.00	0.0619	0.0601	3.00
1.173	0.0594	0.0581	2.24	0.0577	0.0582	−0.86	0.0604	0.0585	3.25
1.333	0.055	0.0545	0.92	0.0553	0.0545	1.47	0.0561	0.0548	2.37
1.408	0.054	0.0529	2.08	0.0523	0.053	−1.32	0.0542	0.0533	1.69

**Table 4:** Effective atomic number (Z_eff_) and equivalent atomic number (Z_eq_) for the granite sample.

E (MeV)	Z_eff_			Z_eq_		
	G.G	G.WH	G.RA	G.G	G.WH	G.RA
0.015	13.64	13.68	13.45	12.31	12.23	12.21
0.02	13.55	13.60	13.35	12.40	12.32	12.28
0.03	13.09	13.13	12.90	12.49	12.43	12.37
0.04	12.47	12.48	12.31	12.54	12.50	12.42
0.05	11.89	11.88	11.77	12.58	12.54	12.46
0.06	11.45	11.42	11.35	12.61	12.58	12.48
0.08	10.92	10.86	10.85	12.65	12.63	12.52
0.10	10.66	10.59	10.61	12.68	12.66	12.54
0.15	10.42	10.34	10.39	12.72	12.72	12.58
0.20	10.35	10.27	10.32	12.75	12.75	12.60
0.30	10.30	10.22	10.28	12.79	12.80	12.63
0.40	10.29	10.21	10.27	12.80	12.82	12.65
0.50	10.29	10.20	10.26	12.81	12.83	12.65
0.60	10.28	10.20	10.26	12.82	12.84	12.66
0.80	10.28	10.20	10.26	12.82	12.84	12.66
1.00	10.28	10.19	10.26	12.82	12.84	12.66
1.50	10.28	10.20	10.26	11.65	11.55	11.57
2.00	10.29	10.20	10.27	11.39	11.27	11.33
3.00	10.32	10.23	10.30	11.33	11.21	11.27
4.00	10.36	10.27	10.33	11.30	11.18	11.25
5.00	10.40	10.31	10.37	11.29	11.17	11.24
6.00	10.44	10.34	10.41	11.29	11.17	11.24
8.00	10.51	10.41	10.48	11.28	11.16	11.23
10.00	10.57	10.47	10.54	11.28	11.15	11.23
15.00	10.69	10.59	10.66	11.28	11.15	11.22

## Data Availability

The data presented in this study are available on request from the corresponding author.
